# In Vitro Evaluation of Aerosol Therapy with Pentamidine-Loaded Liposomes Coated with Chondroitin Sulfate or Heparin for the Treatment of Leishmaniasis

**DOI:** 10.3390/pharmaceutics15041163

**Published:** 2023-04-06

**Authors:** Lucía Román-Álamo, Mohamad Allaw, Yunuen Avalos-Padilla, Maria Letizia Manca, Maria Manconi, Federica Fulgheri, Jorge Fernández-Lajo, Luis Rivas, José Antonio Vázquez, José Esteban Peris, Xavier Roca-Geronès, Srisupaph Poonlaphdecha, Maria Magdalena Alcover, Roser Fisa, Cristina Riera, Xavier Fernàndez-Busquets

**Affiliations:** 1 Barcelona Institute for Global Health (ISGlobal), Hospital Clínic-Universitat de Barcelona, Rosselló 149-153, 08036 Barcelona, Spain; 2 Nanomalaria Group, Institute for Bioengineering of Catalonia (IBEC), The Barcelona Institute of Science and Technology, Baldiri Reixac 10-12, 08028 Barcelona, Spain; 3 Nanoscience and Nanotechnology Institute (IN2UB), University of Barcelona, Martí i Franquès 1, 08028 Barcelona, Spain; 4 Department of Life and Environmental Sciences, University of Cagliari, University Campus, S.P. Monserrato-Sestu Km 0.700, 09042 Monserrato, Italy; 5 Centro de Investigaciones Biológicas Margarita Salas, Consejo Superior de Investigaciones Científicas (CSIC), Ramiro de Maeztu 9, 28040 Madrid, Spain; 6 Group of Recycling and Valorization of Waste Materials (REVAL), Marine Research Institute (IIM-CSIC), Eduardo Cabello 6, 36208 Vigo, Spain; 7 Department of Pharmacy and Pharmaceutical Technology, University of Valencia, 46100 Burjassot, Spain; 8 Section of Parasitology, Department of Biology, Health and Environment, Faculty of Pharmacy and Food Science, University of Barcelona, Av. Joan XXIII 27-31, 08028 Barcelona, Spain

**Keywords:** *Leishmania infantum*, *Leishmania pifanoi*, leishmaniasis, pentamidine, liposomes, drug encapsulation, aerosol therapy

## Abstract

The second-line antileishmanial compound pentamidine is administered intramuscularly or, preferably, by intravenous infusion, with its use limited by severe adverse effects, including diabetes, severe hypoglycemia, myocarditis and renal toxicity. We sought to test the potential of phospholipid vesicles to improve the patient compliance and efficacy of this drug for the treatment of leishmaniasis by means of aerosol therapy. The targeting to macrophages of pentamidine-loaded liposomes coated with chondroitin sulfate or heparin increased about twofold (up to ca. 90%) relative to noncoated liposomes. The encapsulation of pentamidine in liposomes ameliorated its activity on the amastigote and promastigote forms of *Leishmania infantum* and *Leishmania pifanoi*, and it significantly reduced cytotoxicity on human umbilical endothelial cells, for which the concentration inhibiting 50% of cell viability was 144.2 ± 12.7 µM for pentamidine-containing heparin-coated liposomes vs. 59.3 ± 4.9 µM for free pentamidine. The deposition of liposome dispersions after nebulization was evaluated with the Next Generation Impactor, which mimics human airways. Approximately 53% of total initial pentamidine in solution reached the deeper stages of the impactor, with a median aerodynamic diameter of ~2.8 µm, supporting a partial deposition on the lung alveoli. Upon loading pentamidine in phospholipid vesicles, its deposition in the deeper stages significantly increased up to ~68%, and the median aerodynamic diameter decreased to a range between 1.4 and 1.8 µm, suggesting a better aptitude to reach the deeper lung airways in higher amounts. In all, nebulization of liposome-encapsulated pentamidine improved the bioavailability of this neglected drug by a patient-friendly delivery route amenable to self-administration, paving the way for the treatment of leishmaniasis and other infections where pentamidine is active.

## 1. Introduction

*Leishmania* is a kinetoplastid protozoan responsible for leishmaniasis, a vector-borne disease transmitted to vertebrates through the bite of infected female sandflies [1]. This parasite has a digenic life cycle consisting of the flagellated promastigote, dwelling in the gut of the sandfly, which once transmitted into the vertebrate host invades macrophages, where it transforms into the amastigote form that reproduces inside a parasitophorous vacuole [2], the pathological form in vertebrates. Leishmaniasis ranks among the most important tropical neglected diseases, with a wide geographical distribution and an important impact on global health and economic concerns involving humans, domestic animals and wildlife of endemic areas [3]. From clinical criteria, leishmaniasis is classified under three major groups: cutaneous (CL), mucocutaneous (MCL) or visceral (VL). An annual incidence of 600,000 to 1 million cases for CL and 50,000 to 90,000 for VL has been estimated, mostly affecting populations from low- and middle-income countries [4]; about 95% of CL cases occur in the Americas, the Mediterranean basin, the Middle East and Central Asia [5]. In addition to thermo- and cryotherapy and photodynamic therapy for nondiffuse forms of CL [6], chemotherapy is nowadays the only available treatment for leishmaniasis [7,8], but only four drugs are under current clinical use and they are threatened by rising resistance and, for some of them, important side effects and unaffordable cost.

For over 70 years, pentavalent antimonials (i.e., sodium stibogluconate and meglumine antimoniate) have been the first choice in therapy for leishmaniasis, and they still remain so in New World MCL and VL [9]. Although their use for VL is under progressive decline due to severe side effects and rising resistance emergence, even in untreated patients in the Indian subcontinent [10,11], local intralesional pentavalent antimonial injections are the recommended treatment for CL [12], despite being painful and not always effective [13]. The polyene amphotericin B (AmB) has been widely employed as a deoxycholate suspension for VL. Nevertheless, the frequently associated nephrotoxicity has limited its clinical use, a drawback absent in its liposomal formulations (L-AmB) [14]. AmBisome^®^ (Gilead, Foster City, CA, USA), a liposome suspension administered through slow intravenous infusion, is the most used formulation of L-AmB for VL treatment [15,16,17]. Miltefosine (hexadecylphosphocholine), so far the only oral drug against *Leishmania*, has evolved to a first-line medicine since its introduction 20 years ago [18]. However, it is potentially teratogenic and should not be taken by women of child-bearing age without contraception treatment [19]. Paromomycin (aminosidine) is an aminoglycoside antibiotic usually administered intramuscularly to treat VL and CL, with mild pain at the injection site and the ototoxicity associated to aminoglycosides as its most common adverse effects [20]. It is also used as a local treatment in nondiffuse CL as an ointment on the ulcers [12]. Systemic drugs, especially AmB-loaded liposomes, are also used to treat mucocutaneous, diffuse cutaneous and post-kala-azar dermal leishmaniases, but the treatment options are still unsatisfactory [21]. Combination therapies offer an interesting alternative to monotherapy, since most combinations allow to reduce the effective drug dose, cost and time of treatment, in addition to improving the parasitological control of *Leishmania* [22].

**Figure 1:**
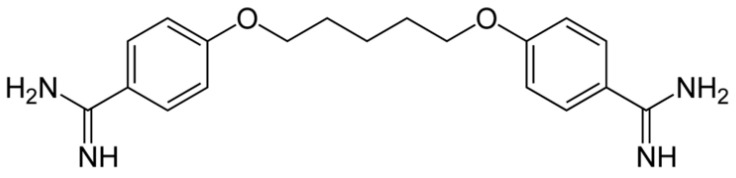
Chemical structure of pentamidine (1,5-bis(4-amidinophenoxy)pentane).

Nowadays, pentamidine (Figure 1), formulated as isethionate, is mostly used as a second-line drug, once failure of or relapse after first-line drugs occurs [23,24], but also extensively employed as a first choice for MCL treatment in many South American regions endemic for this form and for CL caused by *Leishmania guyanensis* and *Leishmania panamensis* (reviewed in [25]). Its use in canine leishmaniasis was also reported [26,27], as well as its role as a component within combination therapies [7,28,29]. The clinical use of pentamidine as leishmanicidal drug has undergone multiple ups and downs, following the effectiveness and availability of other alternative treatments, and has been stalled by its occasional but sometimes irreversible side effects, such as diabetes mellitus, severe hypoglycemia, shock, myocarditis and renal toxicity [30]. Due to its two basic amidine groups, pentamidine isethionate is poorly absorbed in the gastrointestinal tract after oral intake. Thus, for clinical purposes, it is usually administered parenterally, which provides a rapid absorption from the moment of injection and ensures a constant plasma concentration during the first 24 h [31].

To overcome the aforementioned drawbacks of the current chemotherapy against leishmaniasis, it is urgent to develop alternative and innovative treatments, with a special focus on self-administering therapies, drug combinations and different intake routes, achieved through new nanotechnological delivery systems [30,32,33]. The encapsulation of pentamidine has been carried out in several nanodevices such as niosomes coated with chitosan glutamate [34], which is an example of a smart system capable of crossing the blood–brain barrier upon nasal administration, chitosan nanoparticles enriched with mucin as a system for the local treatment of CL [35], or cerium-doped nanoparticles specifically designed for the treatment of VL, thanks to the presence of a polycationic branched polyethylenimine polymer [36]. The delivery of drugs by oral aerosols is more patient-friendly than parenteral administration and profits from the large surface of lung alveoli for adsorption. If a systemic level of pentamidine by aerosol route similar to that obtained by its parenteral administration could be achieved, avoidance of patient hospitalization might be possible [37,38]. The systemic distribution of pentamidine after aerosolization may be ameliorated by its loading into nanocarriers specifically tailored to be effectively nebulized and capable of reaching the deeper airways [39,40]. Particles ranging from 1 to 5 μm are inhalable, but differences in aerosol characteristics can modulate their regional distribution [41]. A decrease in the size of the aerosolized particles down to 1 or 2 μm improves their deposition within alveolar regions, which are the most favorable sites for systemic absorption of drugs and macrophage uptake [42,43]. Liposomes are ideal carriers for lung administration, and several studies confirmed their promising advantages, as they increase the lung bioavailability of delivered drugs due to a reduction in their clearance rate and an improvement of their uptake by macrophages [44]. Indeed, liposomes undergo passive targeting to macrophages, which represents a useful property, especially in the case of parasites that reside within the endocytic pathway compartments of those cells, such as *Leishmania* [45]. To improve macrophage targeting, liposomes can be easily functionalized with specific ligands, such as glycosaminoglycan molecules like chondroitin sulfate (CS) or heparin. Sugar-grafted liposomes encapsulating pentamidine have been shown to increase the therapeutic efficacy of the drug against experimental leishmaniasis in vivo [46]. CS can bind surface molecules such as CD44 or TLR2 and activate macrophages [47], whereas heparin has been described to bind certain parasites such as *Plasmodium falciparum* [48] and *Leishmania chagasi* [49]. Glycosaminoglycan-binding receptors in *Leishmania* include heparin-binding proteins (HBP), and it has been observed that promastigotes incubated with heparin before macrophage infection reached a higher infection rate, suggesting that heparin could play a role in the interaction between HBPs and macrophages [50].

Under these premises, the aim of the present work was to develop an innovative delivery system of low toxicity by loading pentamidine in liposomes coated with CS or heparin specifically tailored for lung administration. The main physicochemical characteristics (mean diameter, surface charge and stability on storage) and technological properties (encapsulation efficiency, aerodynamic behavior and drug release) of different pentamidine-containing nanoformulations have been evaluated along with their toxicity in an endothelial cell line. Finally, the ability of encapsulated pentamidine to inhibit the growth of *Leishmania infantum* and *Leishmania pifanoi* promastigotes and amastigotes was determined and compared with the free drug in solution.

## 2. Materials and Methods

### 2.1. Materials

Enriched soy phosphatidylcholine (Phospholipon^®^ 90G, P90G) was kindly supplied by AVG S.r.l. (Garbagnate Milanese, Milan, Italy). CS extracted from rabbitfish (*Chimaera montrosa*) cartilages was kindly supplied by the Group of Recycling and Valorisation of Waste Materials (REVAL) from the Marine Research Institute (IIM-CSIC, Vigo, Spain) and was produced following the protocol reported in [51]. Pentamidine isethionate, heparin and all other chemicals and solvents of analytical grade were purchased from Sigma-Aldrich (Milan, Italy), unless otherwise indicated. Reagents and plastic labware for cell culture were purchased from Life Technologies Europe (Monza, Italy).

### 2.2. Liposome Preparation and Pentamidine Quantification

To prepare the vesicles (Table 1), P90G (60 mg/mL), with or without pentamidine isethionate (5 mg/mL), was weighed in a glass vial and hydrated with water to a final volume of 1 mL for plain liposomes and 0.9 mL for CS- and heparin-coated liposomes (CS- and heparin-liposomes, respectively). The resulting dispersion was sonicated 2 times (5 cycles, 5 sec on and 2 sec off and 13 µm of probe amplitude) using a high-intensity ultrasonic disintegrator (Soniprep 150, MSE Crowley, London, UK). To prepare CS and heparin liposomes, 100 µL of a water solution of CS (5 mg/mL) or heparin (10 mg/mL) was added dropwise to liposome dispersions. After ultracentrifugation (150,000× *g*, 4 °C, 1 h) and three washes with phosphate-buffered saline, pH 7.4 (PBS), the amount of CS and heparin attached to liposomes was quantified in triplicate assays with the colorimetric Alcian blue assay [52]. Rhodamine-labeled liposomes were prepared as described above, but with a molar lipid formulation P90G:1,2-dipalmitoyl-*sn*-glycero-3-phosphoethanolamine-N-(lissamine rhodamine B sulfonyl) 99.75:0.25. The liposome dispersions were sterilized by filtration through a 0.22 µm filter (VWR, Radnor, PA, USA) under manual pressure.

The quantification of pentamidine was carried out by ultraperformance liquid chromatography (UPLC, ACQUITY H-class Plus system, Waters Corporation, Milan, Italy) using a chromatograph equipped with a UV photodiode array detector and a C18 reverse-phase column (Waters Corporation, 1.7 μm, 2.1 mm × 50 mm) at 25 °C. The composition of the mobile phase was methanol:acetonitrile:solution A (25:10:65% volume). Solution A was an aqueous solution of triethylamine and H_3_PO_4_ (both 0.5% w/vol), adjusted at pH 3.3 with HCl. Flow rate was 200 µL/min for 10 min, and pentamidine, with a retention time of 5.7 min, was detected at 270 nm. A calibration curve was built using pentamidine at different concentrations (0.2, 0.1, 0.05, 0.025 and 0.0125 mg/mL).

### 2.3. Characterization of Liposomes

The average diameter and polydispersity index of liposomes were measured by dynamic light scattering in a Zetasizer Ultra equipment (Malvern Instruments, Worcestershire, UK). Samples were backscattered by a helium–neon laser (633 nm) at an angle of 173° and a constant temperature of 25 °C. Zeta potential was estimated using the Zetasizer Ultra mixed-mode measurement phase analysis light scattering (M3-PALS) configuration, which measures particle electrophoretic mobility. Before the measurements, samples were diluted 1/100 with water. The liposome dispersions were separated from the unencapsulated drug by dialysis. Each sample (1 mL) was loaded into Spectra/Por^®^ polycarbonate tubing (12–14 kDa cut-off, 3 nm pore size; Spectrum Laboratories Inc., Breda, The Netherlands) and dialyzed against water (2 L) at room temperature for 2 h. The pentamidine content was measured by UPLC as reported above after disruption of liposomes with methanol (1:100 dilution). Encapsulation efficiency (EE) was calculated as the percentage of pentamidine recovered after dialysis relative to the amount initially present. The stability of liposome dispersions prior to dialysis was evaluated after their storage at 4 °C for up to 6 months by measuring their mean diameter, polydispersity index and zeta potential at scheduled times.

For cryogenic transmission electron microscopy (cryo-TEM) analysis, 1.5 μL of sample diluted tenfold in water was applied on the carbon surface of a glow-discharged Lacey Carbon 300 mesh copper grid (Ted Pella, Inc., Redding, CA, USA). The sample was kept at 100% humidity inside the chamber of a Vitrobot Mark III (FEI Company, Eindhoven, The Netherlands). The excess of liquid was automatically blotted with filter paper, followed by cryo-immobilization by plunge freezing in liquefied ethane. The plunge-frozen sample was transferred to a Tecnai F20 electron microscope (FEI Company) in the Cryomicroscopy Unit from the Scientific and Technological Centers from the *Universitat de Barcelona* using a cryo-holder (Gatan, Pleasanton, CA, USA). The sample was examined in cryogenic conditions at 200 kV and using low-dose imaging conditions. Images were recorded with a 4096 × 4096 pixel CCD Eagle camera (FEI Company).

### 2.4. Drug Release Analysis

The amount of pentamidine released from the liposomes was measured using a dissolution tester equipped with 6 stations (DT 720 Series-ERWEKA, distributed by EMME 3 SRL, Milan, Italy), using as reference a pentamidine water solution. Pentamidine formulations (1 mL) were transferred into Spectra/Por^®^ dialysis tubes that were placed in the baskets of the dissolution tester containing 1 L of PBS and left under constant stirring at 37 °C, replacing the buffer at different time points (0.25, 0.5, 1, 2, 4, 8, 24, 36 and 96 h). At each time point, 50 µL samples were withdrawn, diluted with 950 µL of methanol:water (1:1), and analyzed for drug quantification. The fraction of released pentamidine was calculated according to the following formula:

$$Pentamidine\;release\left(\%\right)=\frac{released\;pentamidine}{initial\;pentamidine}\times 100$$

### 2.5. Nebulization of Formulations for the Determination of Aerodynamic Behavior

The in vitro deposition of vesicle dispersions was evaluated using the Next Generation Impactor (Eur. Ph 7.2, Copley Scientific Ltd., Nottingham, UK) and the PARI SX^®^ air jet nebulizer connected to a PARI BOY SX^®^ compressor (PARI GmbH, Starnberg, Germany) [53]. Dispersions (2 mL) were placed in the jet nebulizer and aerosolized to dryness directly into the throat of the impactor. At the end of the experiment, the sample deposited into the different stages of the impactor was recovered with methanol, and drug content was quantified by UPLC as reported above. Deposition performances were evaluated calculating the total mass output (TMO), fine particle dose (FPD) and fine particle fraction (FPF). Mass median aerodynamic diameter (MMAD) and geometric standard deviation values were calculated, avoiding the inclusion of the mass deposited in the induction port [54]. The cumulative amount of particles with a diameter smaller than the stated size of each stage was plotted as a percentage of recovered drug versus the cut-off diameter, and the MMAD of the particles was extrapolated from the graph.

### 2.6. Promastigote Growth Inhibition Assay

*L. infantum* promastigotes (strain MHOM/ES/2016/CATB101, isolated from a patient with cutaneous and visceral leishmaniasis [55]), were maintained at 26 °C in Schneider’s insect medium, supplemented with 20% heat-inactivated fetal bovine serum (FBS), 25 μg/mL gentamycin and 1% sterile human urine, pH 6.7 (complete Schneider). Ten 1:2 serial dilutions in complete Schneider of pentamidine (starting from 100 µM), either free or loaded in liposomes, were performed in 96-well microtiter plates (Corning^®^, St. Louis, MO, USA), to which was added 100 µL/well of a suspension of 2 × 10^6^ *L. infantum* promastigotes/mL at their logarithmic growth phase (200 µL/well final volume). After 48 h of incubation at 26 °C, 0.0125% resazurin sodium salt (Sigma-Aldrich Corporation, St. Louis, MO, USA) was added, and plates were incubated for 24 h in the same conditions. Afterwards, resorufin fluorescence (λex/em: 535/590 nm) was measured in a Synergy™ HTX Multi-Mode microplate reader (Thermo Electron Corporation, Waltham, MA, USA).

*L. pifanoi* promastigotes of the MHOM/VE/60/Ltrod line were grown at 26 °C in Roswell Park Memorial Institute 1640 medium (RPMI) supplemented with 10% heat-inactivated FBS plus 2 mM L-glutamine. For cytotoxicity assays, parasites were harvested at late exponential phase of growth and resuspended at 2 × 10^6^ cells/mL in the same medium devoid of phenol red. Parasites were incubated for 72 h with the corresponding test samples disposed in six 1:2 serial dilutions (starting from 25 µM pentamidine). Cytotoxicity was evaluated by the inhibition of MTT (3-(4,5-dimethyl-2-thiazolyl)-2,5-diphenyl-2*H*-tetrazolium bromide) reduction (0.5 mg/mL final concentration) by measuring the reduced formazan, after its solubilization with 0.1% SDS, at 595 nm in a Bio-Rad microplate reader.

For both species, the concentration inhibiting 50% of parasite growth (IC_50_) was determined by nonlinear regression analysis with GraphPad Prism 8.0 (GraphPad Software, La Jolla, CA, USA). Experiments were performed in triplicate.

### 2.7. Amastigote Growth Inhibition Assay

Two complementary methods, microscopic observation and a fluorescence-based technique, were used to evaluate the effect of pentamidine-loaded liposomes on *L. infantum* amastigotes. For preliminary microscopic analysis, 300 µL of murine RAW 264.7 macrophages (Abelson murine leukaemia virus-induced tumour cell line) was seeded (5 × 10^4^ cells/mL) in 8-well chamber slides (ibidi GmbH, Gräfelfing, Germany) and grown in RPMI supplemented with 10% FBS and 1% penicillin–streptomycin (complete RPMI) at 37 °C in the presence of 5% CO_2_ for 24 h, when >90% of macrophages were adhered in each well. Afterwards, infection was allowed to proceed for 24 h. For that, a late stationary phase *L. infantum* promastigote suspension (5 × 10^5^ promastigotes/mL in complete RPMI) was added to each well. Nonphagocytosed parasites were removed by washing, seven 1:2 serial dilutions of pentamidine-loaded liposomes or free drug (starting from 100 µM) were added and macrophages were incubated in the same conditions for an additional 48 h. Then, cells were washed, fixed with 100% methanol and stained with 10% Giemsa for 7 min. IC_50_ was determined by microscopic counting of infected macrophages in a total of at least 300 macrophages per well [56] in triplicate.

To better quantify the preliminary microscopic results, a fluorescence-based approach was used, following a parasite rescue and transformation assay protocol [57] and the resazurin method described above. RAW 264.7 cells were seeded at 10^5^ cells/mL in 96-well microtiter plates and incubated for 24 h at 37 °C in the presence of 5% CO_2_ in order to allow cell adherence. Then, infection was carried out adding *L. infantum* promastigotes at 10^6^ parasites/mL in complete RPMI (1:10 macrophage:parasite ratio). After a further 24 h incubation in the same conditions, nonphagocytosed parasites were removed by washing, and eight 1:2 serial dilutions of drug in solution or loaded in liposomes (starting from 100 µM) were added to each well, and the plates were incubated for another 48 h. Then, cells were washed with FBS-free Schneider’s medium and treated with 40 µL of Schneider’s medium supplemented with 0.05% SDS in order to induce cell lysis. After 40 s, SDS was quenched with 160 µL/well of complete Schneider, plates were incubated for 72 h at 26 °C, and parasite proliferation and the resulting IC_50_ were determined by the resazurin method as described above.

The axenic *L. pifanoi* amastigote line MHOM/VE/60/Ltrod, which affords the study of the amastigote form of this species without interference of the macrophage, was maintained at 32 °C in M199 medium supplemented with 20% heat-inactivated FBS, 0.01% hemin, 0.5% trypticase, 0.25% D-glucose, and 2 mM L-glutamine, pH 6.5. Axenic amastigotes (2 × 10^6^ cells/mL) were incubated with the corresponding test samples (six 1:2 serial dilutions, 25 µM being the highest pentamidine concentration) for 72 h in 96-well plates (200 µL/well). Afterwards, the medium was removed by washing the parasites twice with 1 mL Hank’s medium supplemented with 10 mM D-glucose. Then, MTT reduction by axenic amastigotes was carried out by resuspending them into 100 µL of the same medium containing 0.5 mg/mL of MTT, followed by incubation at 32 °C for 2 h. The formed formazan was evaluated as for promastigotes of the same species, as described above, thus avoiding the autofluorescence and scattering problems that might be encountered with the resazurin method at high concentrations. IC_50_ was calculated with GraphPad Prism 8.0 fitting the data with a nonlinear regression analysis. Experiments were performed in triplicate.

### 2.8. Cytotoxicity Assay

Human umbilical vein endothelial cells (HUVECs) were maintained in M199 supplemented with 10% FBS and 1% penicillin–streptomycin at 37 °C in the presence of 5% CO_2_ and seeded in 96-well plates at a density of 5 × 10^4^ cells/mL. After allowing cell adherence for 24 h, the medium was removed, and eight 1:2 serial dilutions of pentamidine in solution or loaded in liposomes (starting from 500 µM) was added in M199 supplemented with 1% penicillin–streptomycin, and cultures were incubated in the same conditions for 24 h. Then, the medium in each well was replaced with 100 µL of M199 containing 1% penicillin–streptomycin and 10 µL of 4-[3-(4-iodophenyl)-2-(4-nitrophenyl)-2*H*-5-tetrazolio]-1,3-benzene disulfonate labeling reagent (WST-1, Roche, Basel, Switzerland). Plates were incubated for 4 h, and absorbance was measured at 440 nm in a Synergy™ HTX Multi-Mode microplate reader. The concentration inhibiting 50% of cell viability (CC_50_) was determined by linear regression analysis calculated with GraphPad Prism 8.0. Experiments were performed in triplicate.

### 2.9. Confocal Fluorescence Microscopy

RAW 264.7 macrophages were seeded (50,000 cells/mL) in an 8-well chamber slide system (ibidi GmbH) and allowed to adhere overnight. Then, they were incubated with pentamidine-containing, rhodamine-labeled liposomes (55 µM P90G) for 3 h in RPMI. After three RPMI washes, cells were stained for 30 min with 2 μg/mL of the DNA dye Hoechst 33342 and rinsed with RPMI. Fluorescence microscopy analysis was conducted in a Leica TCS SP5 confocal microscope (Leica Camera, Mannheim, Germany). Hoechst 33342 was excited with a 405 nm diode laser and rhodamine with a 561 nm diode-pumped solid-state laser. Fluorescence emissions were collected in the 416–464 and 575–661 nm ranges, respectively.

### 2.10. Flow Cytometry Analysis

A total of 1.5 × 10^5^ RAW 264.7 cells/mL was seeded in T-25 flasks (SPL, Pochon, Kyonggi-do, Republic of Korea) and left overnight to allow their adhesion. Pentamidine-containing, rhodamine-labeled liposomes (165 µM P90G) were then added to each flask and incubated for 3 h with the cells before treating with 2 μg/mL Hoechst 33342 for 30 min. Cells were detached with 0.25% trypsin–EDTA (Sigma-Aldrich Corporation) and the trypsin reaction was stopped by adding 10 volumes of prewarmed RPMI, followed by 3× washes with PBS. The uptake of labeled liposomes was analyzed with a five-laser LSRFortessa flow cytometer (BD Biosciences, San Jose, CA, USA) in the 20-parameter standard configuration. Lasers used for the excitation of Hoechst 33342 and rhodamine were, respectively, 350 and 561 nm, and emissions were collected with 450/50 BP and 570LP-582/15 BP nm bandpass filters. A total of 20,000 events were recorded for each sample.

### 2.11. Statistical Data Analysis

The results are expressed as mean values ± standard deviations, unless otherwise indicated. Statistically significant differences were determined using the one-way analysis of variance and Student’s *t* test. The minimum significance level chosen was *p* < 0.05.

## 3. Results

### 3.1. Liposome Characterization

Empty liposomes, uncoated or coated with CS or heparin, had diameters between 80 and 90 nm and a polydispersity index between 0.30 and 0.36 (Table 2). The encapsulation of pentamidine did not significantly change their mean diameter but led to a slight decrease in the polydispersity index to below 0.30.

The functionalization of empty liposomes with CS and heparin led to a significant decrease in zeta potential, consistent with the coating of liposomes with the negatively charged glycosaminoglycan polymers. On the contrary, pentamidine-containing liposomes had a large positive zeta potential, indicative of the presence in their formulation of the drug, whose two amidine groups impart a strong cationic character. The positive surface charge may be connected with the location of part of the drug on the vesicle surface, especially the positively charged groups. The zeta potential of liposomes encapsulating pentamidine was not significantly affected by CS or heparin addition. The amount of CS and heparin in pentamidine-containing liposomes (60 mg lipid/mL) was, respectively, 0.33 ± 0.04 and 0.36 ± 0.07 mg/mL, as determined by the Alcian blue assay. The encapsulation efficiency of pentamidine was ca. 47%, without statistical differences among the three formulations (*p* > 0.05).

**Figure 2:**
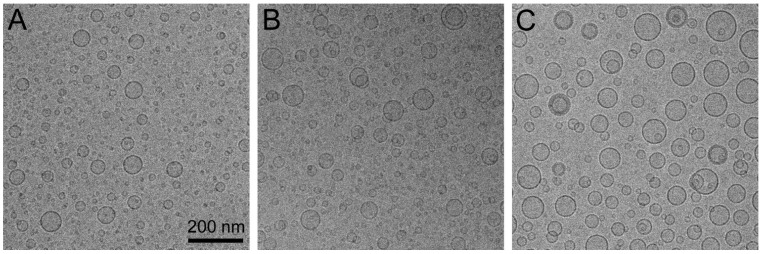
Cryo-TEM images of pentamidine-encapsulating (**A**) plain liposomes, (**B**) CS-liposomes and (**C**) heparin-liposomes, one month after their preparation.

One month after their preparation, pentamidine-containing liposomes were visualized by cryo-TEM to study their morphological characteristics (Figure 2). Cryo-TEM images revealed the presence of mostly spherical unilamellar liposomes with a mean size range below 100 nm, in agreement with dynamic light scattering data.

**Figure 3:**
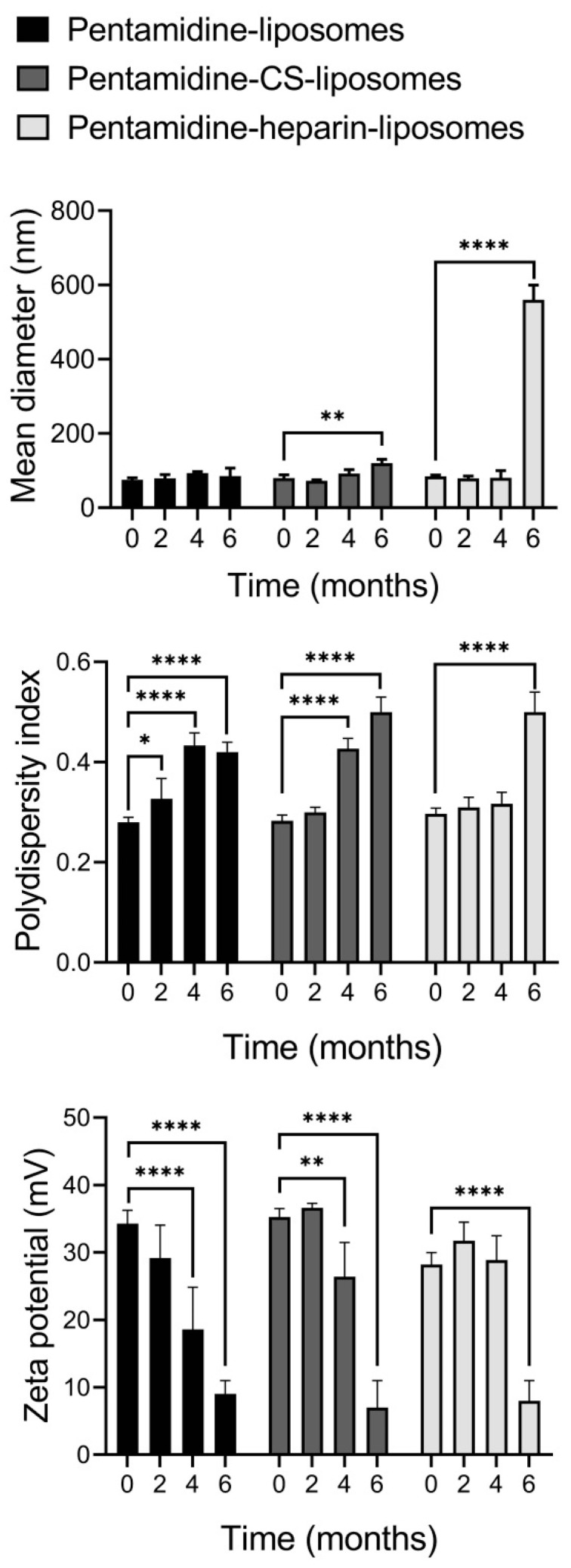
Analysis of mean diameter, polydispersity index and zeta potential of pentamidine-loaded liposomes during 6 months of storage at 4 °C. Mean values ± standard deviations (error bars) are reported (*n* = 3). *: *p* ≤ 0.05; **: *p* ≤ 0.01; ****: *p* ≤ 0.0001.

The physicochemical characteristics of undiluted pentamidine-encapsulating liposome suspensions (containing 60 mg/mL P90G) were evaluated by measuring their mean diameter, polydispersity index and zeta potential at the time of preparation and after 2, 4 and 6 months of storage at 4 °C (Figure 3). While the mean diameter of pentamidine-liposomes did not change significantly, the size of pentamidine-containing CS- and heparin-liposomes underwent a significant increase after 6 months of up to 120 and 500 nm, respectively. The polydispersity index reached values between 0.4 and 0.5 after 4 months for pentamidine- and pentamidine-CS-liposomes, while in pentamidine-heparin-liposomes, it increased above 0.3 only after 6 months. The zeta potential of pentamidine- and pentamidine-CS-liposomes significantly decreased after 4 months, and after 6 months, it reached ~9 mV for all three formulations. This behavior can be due to aggregation and fusion phenomena that may modify the organization of vesicles and also induce the release of the drug or its redistribution in the final system, thus changing the electrical properties of liposome surfaces. Accordingly, for subsequent experiments, all the formulations were used within the first two months after preparation.

**Figure 4:**
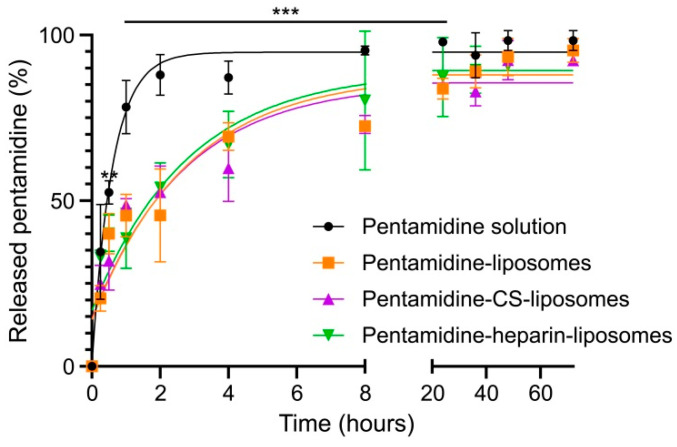
Amount of pentamidine (%) released from dialysis bags along 72 h of incubation in PBS. Mean values ± standard deviations (error bars) are reported (*n* = 3). **: *p* ≤ 0.01; ***: *p* ≤ 0.001 (relative to free pentamidine solution).

The release in PBS of pentamidine from liposomes uncoated and coated with CS or heparin was measured and compared with the free drug in solution using a membrane dialysis approach (Figure 4). Free pentamidine quickly crossed the dialysis membrane, with around 50% of it found in the receptor medium after 30 min and ca. 90% after 2 h, supporting the suitability of this method to evaluate drug release. In contrast, encapsulated pentamidine crossed the dialysis bag at a significantly slower pace, regardless of the used formulation: pentamidine-containing liposomes, either nonmodified or coated with CS or heparin, released ca. 50%, 75% and 90% of drug after 2, 8 and 40 h, respectively, reaching a complete release at 72 h. Because dialysis quickly removes extraliposomal pentamidine, the encapsulated drug exits the liposome to maintain equilibrium, illustrating the contrast between long-term stability of concentrated drug-loaded liposomes and relatively fast content release upon their dilution in aqueous medium.

### 3.2. Cell Targeting of Liposomes

**Figure 5:**
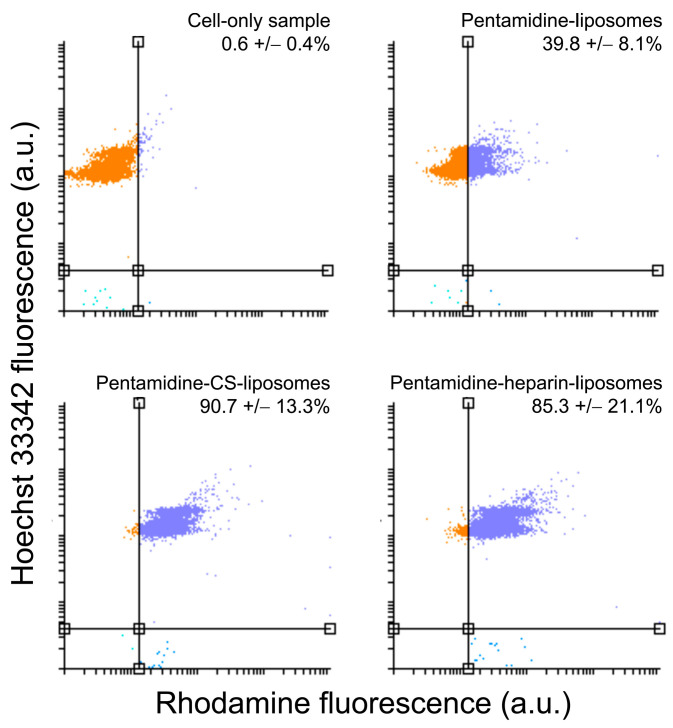
Flow cytometry analysis of liposome targeting to RAW 264.7 macrophages after 3 h of incubation. The vertical line indicates the threshold set to separate rhodamine-free cells (orange dots) from rhodamine-stained cells (purple dots). a.u.: arbitrary units. Mean values ± standard deviations are indicated (*n* = 3).

**Figure 6:**
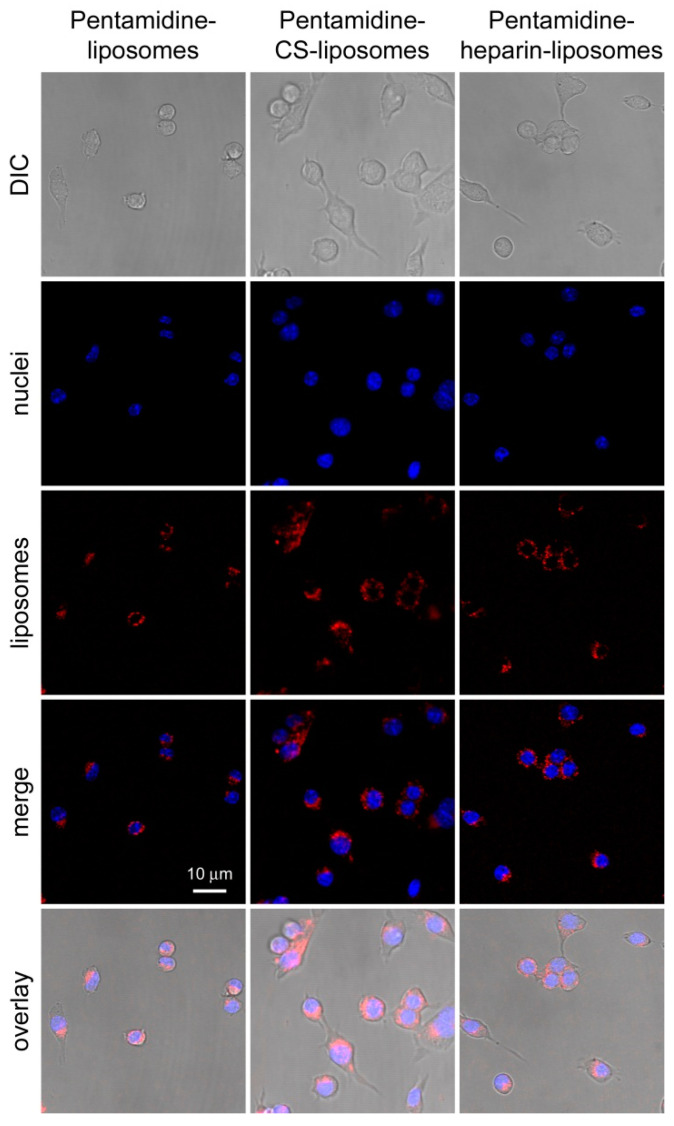
Confocal fluorescence microscopy analysis of pentamidine-loaded liposome uptake in RAW 264.7 macrophages after 3 h of incubation. DIC: differential interference contrast image. Nuclei were stained with Hoechst 33342 and liposomes with rhodamine. Merge: fluorescence channels; overlay: superposition of merge and DIC images.

Targeting to and uptake into RAW 264.7 macrophages of pentamidine-loaded, rhodamine-labeled liposomes was analyzed by flow cytometry and confocal fluorescence microscopy, respectively. After three hours of incubation, pentamidine-liposomes were targeted to ca. 40% of macrophages according to flow cytometry data (Figure 5 and Supplementary Figure S1), whereas this fraction increased to ca. 90% and 85% for pentamidine-CS- and pentamidine-heparin-liposomes, respectively. At this time, the rhodamine label of the formulations was observed inside the macrophages by fluorescence confocal microscopy (Figure 6). The qualitative microscope images actually showed liposome-derived fluorescence in most macrophages for all three formulations. This indicated that the displacement observed for all the cell population in quantitative flow cytometry plots of liposome-containing samples reflected the presence of liposomes in virtually all macrophages, although in some of them their fluorescence was too low to displace them beyond the threshold set by the cell-only sample.

### 3.3. In Vitro Activity of Nanoformulations in Leishmania Promastigotes and Amastigotes

The activity of the different formulations was assayed in *L. infantum* and *L. pifanoi*, commonly causative agents of VL and New World CL, respectively. The IC_50_ for pentamidine on *Leishmania* promastigotes was found to decrease when the drug was encapsulated in liposomes compared with its free form in solution. In the case of *L. infantum* the decrease was significant for all pentamidine-containing liposomes, while in *L. pifanoi*, only when pentamidine-liposomes were coated with CS or heparin (Table 3). All three liposomal formulations of pentamidine had a higher activity against amastigote forms than the free drug, although with modest effectiveness (Table 3 and Supplementary Table S1). The CC_50_ values were always significantly higher for liposome-encapsulated pentamidine, resulting in an improved selectivity index (SI, CC_50_/IC_50_) for all three liposomal formulations relative to the drug in solution (Table 3). The observed accumulation of the liposomes inside macrophages (Figure 6) was consistent with the increased activity on amastigotes of pentamidine-loaded liposomes relative to the free drug. The CC_50_ (HUVECs) and IC_50_ (*L. infantum* promastigotes) of pentamidine-free plain liposomes and liposomes coated with CS or heparin was above the maximum tested concentrations (500 µM and 100 µM, respectively).

### 3.4. Nebulization of Nanoformulations and Aerodynamic Behavior

Several studies indicate that aerosolized liposomes could enhance pulmonary and hepatic delivery and accumulation of entrapped drugs, while minimizing systemic exposure and toxicity [37,58,59]. The deposition of liposome dispersions after nebulization was evaluated using the Next Generation Impactor, which mimics the human airways [60] (Table 4). The nebulization of free pentamidine in solution led to a drug deposition in the deeper stages of the impactor of around 53% and 4 mg (fine particle fraction and dose, respectively), and the median aerodynamic diameter was ~2.8 µm, endorsing only a partial deposition on the lung alveoli. In contrast, nebulization of the dispersions of encapsulated pentamidine increased the fine particle fraction and dose up to ~68% and 5 mg, respectively. The significant increase for encapsulated pentamidine observed in the fine particle fraction (particle percentage with aerodynamic diameter below 5 µm) suggests that liposomes will reach alveoli in a more efficient way than the free drug. Supporting this, the median aerodynamic diameter decreased to between 1.4 and 1.8 µm when pentamidine was encapsulated, indicating a better ability to reach the deeper airways of the respiratory tree in higher amounts, although no statistical differences were found among the three liposome formulations. Taking into account the low mass median aerodynamic diameter of pentamidine encapsulated in liposomes (plain or coated with CS and heparin), these systems seem to be suitable for the treatment of leishmaniasis by aerosol administration.

## 4. Discussion

Current conventional therapies used against leishmaniasis are beset with high toxicity, limited efficacy and high associated cost [61]. Nanotechnology has been implemented in recent years as a promising approach to enhance antileishmanial activity while reducing side effects due to toxicity of the drug [44,62,63].

The location of amastigotes inside macrophage phagolysosomes is responsible for an impaired accession of therapeutic drugs by diffusion across successive membrane barriers, rendering them poorly selective, or forcing their administration in repeated and high doses by parenteral routes [64,65]. These facts contribute to the high toxicity and, in most cases, to the limited efficacy of current conventional medications used against clinical forms of leishmaniasis [66,67,68]. However, the parasitophorous vacuole has selective, yet intense, traffic, with different endosomal compartments. This might render it accessible to drugs vehiculated in nanosystems, profiting from their massive endocytosis routing, and thus endorsing these nanotechnological systems as capable of providing innovative useful tools for future antileishmanial therapies [69]. Among the different nanodelivery systems, liposomes, when administered in vivo by a variety of routes, rapidly accumulate in the mononuclear phagocyte system, a phenomenon profusely used to target drugs for the treatment of intravacuolar parasites that reside in macrophages, such as *Leishmania* [45], as evidenced in the literature [70,71,72,73,74,75,76,77]. Liposomes can be functionalized with polyethyleneglycol covalently attached to phospholipid heads for increased circulation times [78], specific ligands for preferential in vivo delivery to specific cells or organs enriched in the respective receptors or antigens [79], or polymers providing protection from the external environment and increasing mucoadhesion, resulting in an improved residence time of the system (and drug) at the site of action [80]. Because liposome-encapsulated pentamidine will enter the macrophage by endocytosis, and not through receptor-mediated uptake, this will reduce the likelihood of resistance evolution, as it has been shown for African trypanosomiasis, where pentamidine delivered in chitosan nanoparticles bypassed drug resistance mechanisms in the parasite associated to cell surface receptors [81].

The delivery of drugs encapsulated in small liposomes (<100 nm) has been reported to be more efficient than in larger ones, especially when administered in the lungs, likely because of their greater uptake by macrophages [82]. We determined that uncoated liposomes and liposomes coated with chondroitin sulfate or heparin were small in size, homogeneously dispersed and stable up to at least two months of storage at 4 °C. Upon dilution through dialysis, pentamidine release from all three liposome formulations was almost complete (90%) after 40 h, a timeframe that must be taken into account when designing future administration methods and regimens. The current route of pentamidine delivery is intramuscular or intravenous with a regimen of 3 to 20 doses spaced every two or three days depending on the clinical status of the patient [83]. This method is very painful and can lead to side effects associated with the parenteral route, such as muscular aseptic abscess after intramuscular injection [25]. In the present study, nebulization was explored as a harmless route of administration, which would maximize pentamidine deposition in lung alveoli by tailoring liposomes small in size and capable of resisting the harsh conditions that occur during the nebulization process, thus leading to droplets sized between 1 and 2 µm [42,43]. In vitro lung deposition studies underlined the ability of vesicles to deliver pentamidine up to the deeper stages of the impactor in significantly higher amounts than the free compound. However, some nebulized drug may not reach the deeper airways, since a small portion of larger droplets can be produced by the nebulizer, which will interact with the upper part of the respiratory tree where are mainly located the parasites responsible for MCL, the most difficult form of the disease to treat [9]. The nebulization of pentamidine-loaded liposomes is an undemanding methodology and a patient-friendly strategy for the treatment of leishmaniasis, and it is less aggressive than intravenous, intramuscular or subcutaneous administration [84]. The pulmonary delivery of pentamidine can target the drug to *Leishmania* parasites via uptake by the macrophages, reducing toxic systemic effects and limiting the emergence of resistance in the pathogen. If the drug is retained by alveolar macrophages, these cells will behave as a repository for a sustained release of the drug. Alternatively, if the nanocarriers undergo transcytosis and become systemic, they will be available for further uptake by other macrophages. The findings reported here can be especially relevant for the treatment of mucosal leishmaniasis, which damages the upper respiratory tract. As pentamidine is also used for other infections, such as pneumonia produced by *Pneumocystis carinii*, a serious condition that affects people with a weak immune system [85], the spectra of infectious diseases to which nebulization of pentamidine could be applied is not restricted to leishmaniasis.

Although the coating of liposomes with CS or heparin resulted in an enhanced macrophage uptake, this did not translate to a significantly improved IC_50_ for encapsulated pentamidine. It is likely that the relatively fast drug release after its dilution in the cell culture medium, with ca. 50% of it having left the liposomes after 2 h according to PBS dialysis, tends to mask the potential benefits of targeted drug delivery after the 48 h incubation of in vitro growth inhibition assays. Eventual in vivo assays, where the blood residence times will be shortened, might reveal a better performance of the liposomal formulations relative to the free drug. Such targeting to macrophages, underlain by the reported internalization of glycosaminoglycan-coated liposomes through receptor-mediated uptake [47,50], may make them more efficient than uncoated liposomes when tested in vivo. Interestingly, other works documented the induction of an anti-inflammatory status in heparin-treated macrophages [86], which may contribute to reducing the executor function of these cells in synergy with the direct effect of the drug on the parasite, pointing to heparin-coated, pentamidine-containing liposomes as an optimal combination for the treatment of leishmaniasis. Future pharmacokinetics analysis and animal experimentation in preparation for clinical trials should provide valuable information regarding the potential of the nanoformulations developed here to be part of new antileishmanial therapies.

## 5. Conclusions

Given the high toxicity of currently available drugs against leishmaniasis, we undertook a study to ameliorate their therapeutic prospects, using pentamidine as a model compound. The encapsulation of pentamidine in liposomes coated with chondroitin sulfate or heparin provided (i) lower cytotoxicity, (ii) better targeting to macrophages, (iii) a significantly increased efficacy against *L. infantum* and *L. pifanoi* promastigotes and (iv) a higher deposition of the nebulized liposomal preparations in the deeper stages of a human lung airway simulator. Together, these four improvements represent a promising step forward in the formulation of antileishmanial drugs for their optimized administration.

## Figures and Tables

**Table 1:** Composition of liposomes encapsulating pentamidine.

Formulation	P90G (mg/mL)	Pentamidine (mg/mL)	CS (mg/mL)	Heparin (mg/mL)
Pentamidine-liposomes	60.0	5.0	-	-
Pentamidine-CS-liposomes	60.0	5.0	0.5	-
Pentamidine-heparin-liposomes	60.0	5.0	-	1.0

**Table 2:** Mean diameter (MD), polydispersity index (PDI), zeta potential (ZP), and pentamidine encapsulation efficiency (EE) of liposomes. Mean values ± standard deviations are reported (*n* = 6).

Formulations	MD (nm)	PDI	ZP (mV)	EE (%)
Plain liposomes	85 ± 5	0.302 ± 0.002	−9 ± 4	−
CS-liposomes	79 ± 6	0.361 ± 0.006 *	−14 ± 5 **	−
Heparin-liposomes	81 ± 6	0.351 ± 0.004 *	−13 ± 6 **	−
Pentamidine-liposomes	81 ± 3	0.274 ± 0.001	+34 ± 3 ***	47 ± 4
Pentamidine-CS-liposomes	82 ± 4	0.282 ± 0.003	+34 ± 5 ***	48 ± 5
Pentamidine-heparin-liposomes	84 ± 4	0.285 ± 0.002	+35 ± 5 ***	46 ± 6

*: *p* ≤ 0.05; **: *p* ≤ 0.01; ***: *p* ≤ 0.001 (relative to plain liposomes).

**Table 3:** IC_50_, CC_50_ and selectivity index (SI, CC_50_/IC_50_) for pentamidine in solution or loaded in liposomes. Mean values ± standard deviations are reported (*n* = 3).

	CC_50_ HUVECs (µM)	*L. infantum*			*L. pifanoi*		
		IC_50_ Promastigotes (µM)	IC_50_ Amastigotes ^1^ (µM)	SI ^2^	IC_50_ Promastigotes (µM)	IC_50_ Axenic Amastigotes (µM)	SI ^2^
Pentamidine solution	59.3 ± 4.9	25.8 ± 2.2	3.5 ± 0.9	17.2	7.9 ± 3.7	10.7 ± 3.8	5.5
Pentamidine-liposomes	112.0 ± 12.0 ***	11.3 ± 1.7 ***	2.6 ± 0.4	42.9	3.5 ± 1.0	8.3 ± 0.5	13.6
Pentamidine-CS-liposomes	83.4 ± 9.3 *	18.7 ± 3.7 *	2.5 ± 0.4	33.6	2.0 ± 0.4 *	10.2 ± 1.2	8.2
Pentamidine-heparin-liposomes	144.2 ± 12.7 ****	19.7 ± 1.5 *	3.3 ± 0.8	43.3	2.4 ± 0.4 *	9.9 ± 0.7	14.5

^1^ Data obtained with the fluorescence method. The data obtained with the microscopy method are presented in Supplementary Table S1. ^2^ In amastigotes. *: *p* ≤ 0.05; ***: *p* ≤ 0.001; ****: *p* ≤ 0.0001 (relative to pentamidine solution).

**Table 4:** Total mass output (TMO), fine particle dose (FPD), fine particle fraction (FPF) and mass median aerodynamic diameter (MMAD) ± geometric standard deviation (GSD) measured for nebulized pentamidine, free in solution or encapsulated in liposomes. Mean values ± standard deviations are reported for TMO, FPD and FPF (*n* = 3).

	TMO (%)	FPD (mg)	FPF (%)	MMAD (µm) ± GSD
Pentamidine solution	72 ± 6	4.0.± 3.0	53 ± 4	2.8 ± 2.0
Pentamidine-liposomes	74 ± 7	5.0 ± 0.5	70 ± 3 ***	1.7 ± 1.4
Pentamidine-CS-liposomes	82 ± 12	5.0 ± 2.0	68 ± 4 **	1.8 ± 1.5
Pentamidine-heparin-liposomes	84 ± 3 *	5.0 ± 2.0	65 ± 11 *	1.4 ± 1.3

*: *p* ≤ 0.05; **: *p* ≤ 0.01; ***: *p* ≤ 0.001 (relative to pentamidine solution).

## Data Availability

All the data supporting the reported results can be found in the main article and in the Supplementary Materials files.

## Supplementary Materials

The following supporting information can be downloaded at: https://www.mdpi.com/article/10.3390/pharmaceutics15041163/s1, Supplementary Figure S1: One-way analysis of variance to determine statistically significant differences between the different samples of the flow cytometry data in Figure 5; Supplementary Table S1: Comparison of the IC_50_ of pentamidine in *L. infantum* amastigotes calculated with the microscopy and fluorescence methods.
