# Supplementary material for: In Vitro Evaluation of Aerosol Therapy with Pentamidine-Loaded Liposomes Coated with Chondroitin Sulfate or Heparin for the Treatment of Leishmaniasis
Source: Pharmaceutics. 2023 Apr 6;15(4):1163. doi: 10.3390/pharmaceutics15041163 (PMC10147000; doi:10.3390/pharmaceutics15041163)
Supplement: Supplementary file 1 [file pharmaceutics-15-01163-s001.zip › pharmaceutics-2283039-supplementary.pdf]

Supplementary Materials

In Vitro Evaluation of Aerosol Therapy with Pentamidine-loaded Liposomes Coated with Chondroitin Sulfate or Heparin for the Treatment of Leishmaniasis

Lucía Román-Álamo, Mohamad Allaw, Yunuen Avalos-Padilla, Maria Letizia Manca, Maria Manconi, Federica Fulgheri, Jorge Fernández-Lajo, Luis Rivas, José Antonio Vázquez, José Esteban Peris, Xavier Roca-Geronès, Srisupaph Poonlaphdecha, Maria Magdalena Alcover, Roser Fisa, Cristina Riera, and Xavier Fernàndez-Busquets

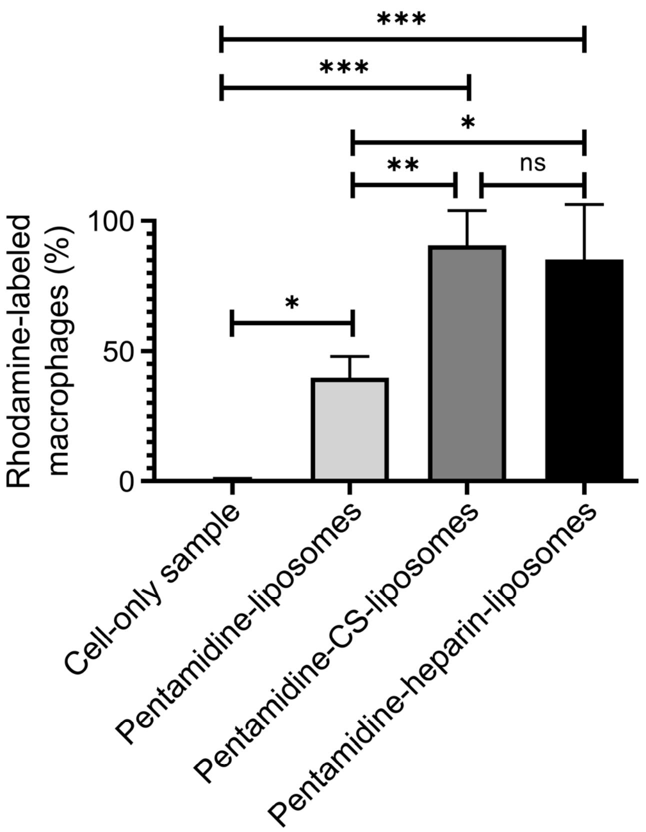

Supplementary Figure S1. One-way analysis of variance to determine statistically significant differences between the different samples of the flow cytometry data in Figure 5. *: $p \leq 0.05$; **: $p \leq 0.01$; ***: $p \leq 0.001$ (relative to the control cell-only sample).

Supplementary Table S1. Comparison of the IC_{50} of pentamidine in *L. infantum* amastigotes calculated with the microscopy and fluorescence methods.

	Pentamidine IC_{50} in *L. infantum* amastigotes (µM)	
	Microscopy method	Fluorescence method
Pentamidine solution	5.6 ± 1.4	3.5 ± 0.9
Pentamidine-liposomes	4.7 ± 2.9	2.6 ± 0.4
Pentamidine-CS-liposomes	3.1 ± 0.6	2.5 ± 0.4
Pentamidine-heparin-liposomes	4.1 ± 0.7	3.3 ± 0.8
